# Disaster recovery and business continuity: A case of MSMEs in Dar es Salaam

**DOI:** 10.4102/jamba.v16i1.1714

**Published:** 2024-10-28

**Authors:** Tumpale Sakijege

**Affiliations:** 1 Department of Urban and Regional Planning, School of Spatial Planning and Social Sciences, Ardhi University, Dar es Salaam, Tanzania

**Keywords:** flood, business recovery, MSMEs, disaster recovery, business recovery, flood, Kigogo

## Abstract

A large number of micro, small and medium enterprises (MSMEs) in developing countries operate in informal settlements. Most of these settlements exist in hazardous environment despite being affected by floods. This research discusses the scarcity of knowledge on the resilience of businesses operating in Kigogo flood-prone informal settlements in Dar es Salaam. It especially focusses on MSMEs. Although recovery of MSMEs has been intensively studied worldwide, in Tanzania it is downgraded. This article is set to bridge this glaring gap focusing on MSMEs recovery after floods. The research methodology employed quantitative research design by completing questionnaires through interviews. A total of 202 owners of MSMEs were interviewed. While content analysis was used to analyse qualitative data, the research further employed a logistic unit model to analyse factors explaining recovery of MSMEs after floods. The study revealed a statistically significant association between the recovery of MSMEs and suitable plans for safeguarding business, personal savings, profit (saving from business) and government support. Other factors include family and friend’s support, business continuity training, longevity in business operation, pre-disaster mitigation measures, critical destruction to business facilities, and running several businesses. Strength, Weaknesses, Opportunities and Threat (SWOT) analysis of the business environment where MSMEs operate revealed a number of issues that inform policymakers, urban planners and environmentalists on what can be done to enhance recovery and hence promote sustainable development.

**Contribution:**

The study adds to the body of knowledge by drawing attention to factors that predict disaster recovery of MSMEs operating in flood-prone informal settlements.

## Introduction

The question of why micro, small and medium enterprises (MSMEs) fail to continue with business, especially after flood disasters, has remained a topical research capturing the attention of various scholars globally (Kato & Charoenrat 2018; Marshall et al. 2015). However, following stiff business competition and the desire to reduce the adverse economic impacts of flood disasters on the residents living in areas affected, various studies have focused on the importance of solving environmental challenges so that those engaged in business can benefit from their efforts (Josephson, Schrank & Marshall 2017). Studies have shown that MSMEs struggle to spend little and hard-to-obtain financial resources to establish business (Samantha 2018). That implies it would be worthwhile to protect their businesses against unforeseen consequences such as flood disasters. Samantha (2018) pointed out that there is a difference between large businesses and small businesses in the question of preparing for any unsuspected threat. He reports that large businesses have more resources that enable them to prepare and plan for the unforeseen, while the managers of small businesses have little understanding and ability to plan for the unanticipated. Over the world, disasters have continued to cause a heavy toll and have caused harm to the well-being of communities, families, individuals, businesses and the country in general. Overall, billions of people have been affected by disaster in different ways and led them to vulnerable situations disproportionately (United Nations 2015). In the year 2021, the world recorded about 432 disasters that are natural hazard related. These disasters accounted for 10 492 deaths, affected about 101.8 million people and caused world economic loss of 252.1 billion US$ (CRED 2022). CRED (2022) pointed further that in 2021, floods overshadowed all disasters with 223 occurrences.

Other authors (Endendijk et al. 2024; Rappaport 2014; Sakai & Yao 2023; Sakijege & Dakyaga, 2023; Rwehumbiza & Sakijege, 2021) pointed out that flooding is among the most costly disasters in the matter of business damage and loss. As a result of the continued presence of climate change, floods are expected to increase rapidly and also the damage they cause is also expected to heighten (Hirabayashi et al. 2013). Wasko (2019) pointed further that climate change has a major impact on changing the pattern of rain and floods. Likewise, Hemmati, Ellingwood and Mahmoud (2020) add that climate change when combined with population and economic growth will steadily increase economic losses in such an environment. This implies that it is urgent and important to anticipate, plan and reduce the risk of disasters in order to effectively protect people, community and their economic assets, thereby strengthening their resilience (United Nations 2015). In disaster studies, it is evident that a number of strategies have been put in place for flood risk reduction; these strategies include investments in structural strategies (e.g. building flood walls) and the use of laws and regulations as methods to sway flood behaviour (Morrison, Westbrook & Noble 2018). However, despite all these efforts to reduce the effects of floods, the losses caused by floods have been increasing progressively. The effects are far greater for those who live in low-lying areas (including businesses); this situation raises many questions about the suitability of strategies used.

Many areas that are subject to frequent floods are often poorly planned and therefore make small-scale businesses operate in a difficult and vulnerable environment (Lundy, Patterson & O’Neill 2017). Flooding brings various effects and causes direct damage to life, buildings and infrastructure. Likewise, flooding can cause financial challenges for businesses and governments in general (Echendu 2020; Merz et al. 2021). Notably, various governments around the world, immediately after the great floods, are obliged to provide financial assistance that can help mitigate the effects of flooding; this kind of assistance may strain limited government resources (Chereni et al. 2020; Liao, Chan & Huang 2019). Disasters, especially floods, reduce the economic progress that has been achieved and create an environment where people are afraid to start a business. According to Husby, Mechler and Jongman (2016), business owners must take into consideration flood risks when they make decisions about what and where to start their businesses. Doing so will reduce the impact if disaster occurs. It is important to emphasise that MSMEs are one of the most trusted groups in contributing to economic growth around the world, as well as contributing significantly to employment and innovation (Ajuwon, Ikhide & Akotey 2017; OECD 2017; Tambunan 2019). In Tanzania, the Ministry of Industry, Trade and Investment (MITI) conducted a baseline survey and published information about MSMEs in 2012. The study showed that until 2010, there were more than 3 million MSMEs in the country. About 94.6% of the MSME were private. The MITI reports estimated that these MSMEs contribute about 27% to GDP (URT 2012). This implies that there is a need to ensure that MSMEs work in a good environment because their presence contributes not only to private welfare of individual citizens but also to the national economy generally.

The current study was founded on the argument that there existed limited research knowledge in Tanzania focusing on MSMEs’ business recovery preparedness. In a stark contrast, the notion of business recovery after flood events has been almost overly studied, particularly in Asian countries such as Thailand, Indonesia, India and Sri Lanka (Josephson et al. 2017; Kato & Charoenrat 2018; Marshall et al. 2015; Sydnor et al. 2017). In the Tanzanian context, studies that exist have tended to focus on the role of saving groups on disaster resiliency (Panman et al. 2022), hybrid governance to disaster management (Clark-Ginsberg, Blake & Patel 2022) and community resilience to natural hazards (Hambati & Yengoh 2018). This research is meant to cover this prevailing gap.

## Literature review

### Conceptual issues: Micro, small and medium enterprises, business continuity and business recovery

Micro, small and medium enterprises are defined as micro enterprises employing less than 5 employees, small-sized enterprises employing less than 20 staff, and medium-sized enterprises employing less than 100 staff (Dewi & Mahendrawathi 2019). Others define MSME based on investment in plant and machinery (Jassim & Khawar 2018). In this study, MSMEs are defined as micro enterprises with employees between 1 and 5. Moreover, they have capital investment in machinery worth up to TZS. 5 million. This implies that any micro business with a capital of more than 5 million will not be counted in the MSME group even if it has between 1 and 5 employees.

Disaster recovery, on the other hand, has different interpretations. It refers to the act of reopening a business immediately after a disaster, regardless of the business’s status (Lam et al. 2012). Disaster recovery involves partnership between two parties with the aim of providing aid and helping disaster victims (Maret et al. 2010). The two parties mentioned here are government and non-government institutions. It is suggested that it is very important for the community to cooperate in the recovery process (Aldrich & Meyer 2015). This means that disaster recovery is all about community participation. Understandably, though, it is suggested that disaster recovery should not be limited to restoring the situation as it was to its original state but should involve future development and business growth after being affected by floods (Pathak & Ahmad 2016).

One of the basic questions meriting contemplation is whether small businesses have the ability to survive. As pointed out by Asgary, Anjum and Azimi (2012), this question is important because small businesses serve the community by creating employment, supplying goods and services and paying tax to the government. However, regardless of the level of business (i.e. international or small businesses), Sahebjamnia, Torabi and Mansouri (2015) emphasise the importance of having a business continuity plan because it helps in protecting activities, personnel, facilities and materials. Blyth pointed out that business continuity plan has three main areas: contingency planning (e.g. establishing mitigation measures to minimise risks), crisis management and recovery (using the methods that were prepared before the disaster to recuperate and return to operations). This explanation implies that business continuity plan and disaster recovery go together. Moreover, many businesses depend on aid from the government and therefore do not have business continuity plans and disaster recovery measures (Paul, Insah & Nangpiire 2014). This situation leads to failure to recover immediately after catastrophe because the help they depend on is not available. Likewise, Chang et al. (2022) identified two factors that cause small businesses to be vulnerable and fail to recover from flood disasters, namely, direct characteristics (e.g. lack of formal disaster plan) and indirect ones (e.g. inexperience and small business size).

### Empirical studies

A study done by Asgary et al. (2012) explored the factors influencing disaster recovery in Pakistan, using a case of small businesses. It was conducted 6 months after the 2010 flood in Pakistan, which severely impacted nine districts. The study findings revealed that 90% of the sample businesses re-opened 6 months after the flood, but majority of them were operating with loss, while only a small number were at the same level or better off. The study indicated the prevalence of a significant relationship between recovery time and average monthly sales or income. The inverse relationship measured by Pearson’s *R* showed that businesses with larger average monthly sale before the flood recovered quickly. The study showed that past disaster experience mattered in influencing the time of disaster recovery. Prior businesses’ disaster experience was linked to faster business recovery. Additionally, government support (Neeraj et al. 2020), business continuity training and/or education and personal savings (Asgary et al. 2012) had the biggest impact on the speed of disaster recovery. Additionally, having multiple locations for similar businesses is an added advantage, as is the size of the business in question and whether the place where the business takes place is owned or rented. A business that has multiple locations may have enough resources to deal with disasters when they occur (Xiao & Van Zandt 2012).

Another study conducted by Khan and Sayem (2013) in flood-prone areas of Bangladesh sought to understand how small businesses recovered from natural hazards. The study was carried out among 251 small-scale business owners using a cross-sectional survey. Convenient sampling was used to obtain the study participants. Other considerations include the characteristics of the businesses, enabling factors and disaster-impact factors that could help explain and identify the elements contributing to business recovery from natural hazards. The study found that those business owners who had higher monthly income were more likely to experience delayed business recovery, the finding that had also been reported by a previous study carried out in the United States (Corey & Deitch 2011). These findings were so striking because one would have expected that those business owners with higher incomes are relatively better positioned to have bank savings or other investments that could help them offset the losses accrued from disaster events. Hence, it could be logical to think that large businesses immediately recover from disaster. The factors attributed to these unexpected findings were as follows: business owners who run a number of businesses possibly continue to do so even if they had been constrained to close down some of the businesses that are operating. In essence, the income obtained from the remaining operating businesses could sustain the family. As such, there would not be an immediate need to recover the affected business. The study revealed that owners with a higher number of years in the business were less likely to experience delayed recovery, while those privileged to have institutional education delayed recovery from disaster.

Khan and Sayem (2013) studied the factors affecting resiliency in small businesses. The researchers found that new businesses were more likely to fail recovering from flood events than those long established. Conversely, the long-established businesses had a relatively higher chance of recovering from disaster. A study has also shown that the longer a business has been in operation, the greater the resilience. Furthermore, Khan and Sayem (2013) found that education had a mixed impact on business resilience. The study found a positive effect on physical capital-based resilience and negative effect on human- and natural capital-based resilience. Khan and Sayem (2013) have added that disaster compelled small businesses to relocate. The finding is amplified by Samantha (2018) who added that considering that disaster causes displacement of the local population and losses among the victims of an affected area, the demand for many products and services is reduced, resulting in small businesses experiencing high rates of failure after disaster.

The literature review revisited has raised several factors that determine business recovery after a flood disaster events. Inferably, large business recovers faster after floods. Moreover, many years of business operation, huge capital investments and high education level are the positive factors for quick recovery of businesses after flood disaster. Another positive factor for business recovery is having in place pre-disaster mitigation measures. Conversely, big loans are negatively linked to business recovery. Nonetheless, the current body of literature is inclined towards studying how large business and SME recover from flood disasters. The current study examined the micro, small-scale dimension. It investigated how MSMEs in the informal settlements of Dar es Salaam recover from flood events. As rightly confirmed by Rodríguez et al. (2007), ‘disaster recovery represents the least understood aspects of emergency management, from the standpoint of both the research community and practitioners’.

## Research methods and design

The study was conducted in Kigogo ward, which has a total of three streets (sub-wards). These are Kigogo Kati, Kigogo Mkwajuni and Kigogo Mbuyuni in Dar es Salaam. Kigogo ward was considered appropriate for the study because it has been experiencing frequent incidences of floods particularly because it is surrounded by rivers (Kibangu, Msimbazi and Tenge rivers; Zugcic 2020).

The data were collected in two different phases. The first phase was a pilot study interview, which involved some MSMEs. The pilot phase was necessary for refining the questions to minimise ambiguities. The pilot study was rationalised by a need to inform the second phase and identify the MSMEs that were most affected by flooding. During the pilot phase, a total of 20 managers of MSMEs were surveyed. In the second phase, a total of 182 managers of MSME were surveyed. The second phase of research was conducted between April and November 2022. In the beginning, the research methodology utilised a mixed-method approach incorporating both qualitative and quantitative data collection. Borrowing from Lundy et al. (2017), ethical issues were observed prior to starting data collection and throughout the period of study. The study employed triangulation methods (Forero et al. 2018) by engaging both the lead researcher and the research assistants, and by integrating diverse data sources, including secondary and primary data, to enhance the consistency and reliability of the research findings.

The research proposal was submitted and approved by the Head of Department of Urban Regional Planning in the Ardhi University. The participants in the study were adults who owned small businesses. Interviews were conducted in Kiswahili as it is a language known by almost all Tanzanians. A total of 202 MSMEs were reached for interviews with the assistance of a research assistant.

Dependent and explanatory variables were used for the purpose of establishing the factors explaining the recovery of MSMEs in flood-prone areas. A number of variables (see Table 1) explaining and influencing business recovery include higher monthly income (Corey & Deitch 2011), longevity of business operation and education (Khan & Sayem 2013), pre-disaster mitigation measures (Tyler & Sadiq 2019), personal savings, business facility, family and friends support, government and non-governmental support (Asgary et al. 2012).

**TABLE 1:** Distribution of businesses’ characteristics.

Variable	Observation	Percentage (%)
**Gender**		
Male	120	59.41
Female	82	40.59
**Education**		
Primary	69	34.16
Secondary	90	44.55
College	39	19.31
University	4	1.98
**Types of business conducted**		
Retail businesses	78	38.61
Food-related business	41	20.30
Service-related business	17	8.42
Others	66	32.67
**Amount of capital invested (TZS)**		
50 000 ≤ *x* ≤ 200 000	56	27.72
200 000 ≤ *x* ≤ 400 000	49	24.26
400 000 ≤ *x* ≤ 600 000	37	18.32
600 000 ≤ *x* ≤ 800 000	15	7.43
800 000 ≤ *x* ≤ 1 000 000	17	8.42
1 000 000 ≤ *x* ≤ 2 000 000	16	7.92
2 000 000 ≤ *x* ≤ 4 000 000	10	4.95
< 5 000 000	2	0.99
**Sources of capital**		
Bank	97	48.02
Friends/family	75	37.13
Personal savings	49	24.26
Saving and lending groups (e.g. Saccos/Vicoba)	30	14.85
Others	5	2.48
**Number of employees**		
0	77	38.12
1	81	40.10
2	25	12.38
3	13	6.44
4	5	2.48
12	1	0.50

TZS, Tanzanian Shilling.

The population size for this study is unknown because there is no record of MSMEs in the informal settlements. Where the population size is unknown, the sample size can be derived by computing the minimum sample size required for accuracy in estimating proportions through considering the standard normal deviation set at 95% confidence level (1.96), percentage picking a choice or response (50% = 0.5) and the confidence interval (0.05 = ±5). The formula used in calculating the minimum sample size (*n*) required is given by *n* = *z^2^*(*P*)(*1–P*)/*e^2^*, where *z* is the value corresponding to the level of confidence required; *P* is the proportion belonging to the specified category, in this case *p* = 0.5; and e is the level of precision. Therefore, the minimum sample size required is 200 respondents. Quantitative data obtained from questionnaire and interview were analysed using the SPSS and STATA. Analysis of qualitative data was done simultaneously with data collection through content analysis (Vaismoradi, Turunen & Bondas 2013). This helped the researcher to refine some of the research questions and consequently led to collection of the meaningful data. Furthermore, the logit model was used to model the recovery of MSMEs after flood and is presented in equation 1:

$$ln\left(\frac{p_{i}}{q_{i}}\right)=\beta_{0}+\sum_{i=1}^{10}\beta_{i}x_{i}+\varepsilon_{i}$$ [Eqn 1]

where:

P_*i*_ = is the probability of the event occurring

q_*i*_ = is the probability of the event not occurring.

### Ethical considerations

Ethical clearance to conduct this study was obtained from the Ardhi University, Department of Urban and Regional Planning (URP) and Department of Economics and Social Studies (ESS) (No. AC. 303/327/01).

## Results

### Business characteristics

Statistically, most of the respondents were men (120) (equal to 59.41%) compared to women (82; i.e. 40.59%). In general, most respondents had attained primary and secondary education as shown in Table 1. Very few (4 out of 202) had university level education. Based on the analysis, respondent’s age has a mean score of 32.9 (s.d. = 5.9). The study found that businesses in informal settlements are dominated by MSMEs. From the analysis, 202 respondents (99.5%) have employees of 0 ≤ *x* ≤ 4, and only one business reported to employ 12 workers.

More than 40 businesses were listed, including fruit selling, food vending, pubs, tailoring and clothing store and pharmacies. To simplify the analysis, all businesses were grouped into four groups: (1) retail-related business; (2) service-related business; (3) food-related business and (4) others. According to the analysis, most fell into retail shops (78:38.61%). Other businesses are small and very ordinary, indicating that they are hand-to-mouth businesses.

When asked about the amount of capital invested in their businesses, the majority (77.73%) reported to have invested the capital that lies between TZS. 50 000 ≤ *x* ≤ 800 000. Given the findings, two impressions are notable: (1) businesses are individual business and (2) they are for subsitence, which are meant for sustenance and meeting other family needs. On the other side, only two respondents (0.99%) had invested TZS. 5 000 000. This amount implies that business in Kigogo is dominated by MSMEs. As discussed by other scholars (Ajuwon et al. 2017; OECD 2017; Tambunan 2019), MSMEs have a great contribution in increasing employment. This study found similar situations for business owners in Kigogo. More than half of the business owners (125; 61.9%) admitted to have hired assistants in their businesses. In addition, more than half (124; 61.4%) admitted to hiring assistants between 1 ≤ *x* < 5.

### Flood impacts on the business

To examine which businesses experienced flood impact, a χ^2^ was carried out. According to the results, there was association between retail-related business and damage to equipment (χ^2^ = 5.1024, *p* = 0.024). Similarly, the findings (Table 2) show significant association between retail business and destruction of business area (χ^2^ = 7.4526, *p* = 0.006).

**TABLE 2:** Association between types of business and flood impact.

Type of business	chi^2^ (*χ*^2^)	*P*	Decision
**Retail-related business**			
Physical injuries	3.1807	0.075	Do not reject Ho
Loss of properties or goods	2.235	0.135	Do not reject Ho
Robbery	0.7313	0.392	Do not reject Ho
Damage of equipment	5.1024	0.024	Reject Ho
Destruction of business area	7.4526	0.006	Reject Ho
**Service-related business**			
Physical injuries	0.3242	0.569	Do not reject Ho
Loss of properties or goods	1.3756	0.241	Do not reject Ho
Robbery	0.2228	0.637	Do not reject Ho
Damage of equipment	0.009	0.925	Do not reject Ho
Destruction of business area	0.2184	0.64	Do not reject Ho
**Food-related business**			
Physical injuries	1.8466	0.174	Do not reject Ho
Loss of properties or goods	9.5398	0.002	Reject Ho
Robbery	1.8452	0.174	Do not reject Ho
Damage of equipment	2.5122	0.113	Do not reject Ho
Destruction of business area	0.0289	0.865	Do not reject Ho

The study found an association between food-related business and loss of properties/goods (χ^2^ = 9.5398, *p* = 0.002). Other groups of business (service oriented) did not experience any flood-related impact.

### Recovery of micro, small and medium enterprises and speed of recovery

It was important to examine if MSMEs recovered after floods and after how many days. The time that each MSMEs had to choose was between 0 and 14 days. The 14 days were opted given the nature of the businesses conducted (majority were hand-to-mouth businesses). So, any business owner who did not fit between the recommended days was considered to have not recovered. On average, 80 MSMEs (equivalent to 39.8%) could not recover. A significant number of MSMEs recovered their businesses; however, the speed of recovery differs. Of 202 MSMEs interviewed, more than half (121; 60.2%) were able to recover their business within 9 days after flooding.

There are many factors that affected the speed of recovery of their business. Slightly more than half (127: 63.18%) of the respondents reported stagnant water as a major factor that caused delay in resuming normal business operations. Others reported serious damage to business facilities (32:15.92), absence of capital to reinvest (27:13.43). On the other hand, other respondents (15) reported other reasons that were not included in the question options; they include fear of not getting customers, destruction of business area and decline in the number of customers.

### Factors explaining recovery of micro, small and medium enterprises

The study applied logistic regression model to explain the factors that explain business recovery. As shown in Table 3, since (*p* = 0.0072) we have enough evidence to reject the null hypothesis at level of significance α = 5% (0.05), hence there is a statistically significant association between business recovery with pre-disaster mitigation measures, suitable plans to safeguard business, profit (saving from business), running several businesses, personal savings, government support, family and friend’s support, business continuity training, longevity in business operation and critical destruction to business facilities.

**TABLE 3:** Logit Regression for Factors influencing recovery of MSMEs after floods.

Recovery	Coefficient	Std. Err.	*z*	*P* > *z*
Pre-disaster mitigation measures	−0.344	0.424	−0.810	0.417
Suitable plans to safeguard business	0.155	0.438	0.350	0.724
Profit (Savings from business)	0.177	0.555	0.320	0.750
Running more than one business	−0.191	0.387	−0.490	0.622
Personal Savings (from other sources)	0.914	0.446	2.050	0.040
Government Support	−1.663	0.477	−3.490	0.000
Family/Friends support	0.591	0.440	1.340	0.179
Business training	−0.413	0.395	−1.050	0.296
Years spent in business	−0.054	0.429	−0.130	0.899
Constant	−0.220	0.645	−0.340	0.732
Number of observations	193	-	-	-
LR Chi^2^	22.58	-	-	-
Prob > chi^2^	0.0072	-	-	-
Pseudo R^2^	0.0886	-	-	-

Std. Err., standard error.

Likewise, it was important to interpret logit using odds ratio, explaining the odds of each independent variable while other variables are constant. A unit increase of pre-disaster mitigation measures increases the odds of recovering to the business by 65.95% compared to those not recovering their businesses. A unit increase of suitable plans to safeguard business decreases the odds of recovering the business by 84.50% compared to those not recovering their businesses. A unit increase in profit (saving from business) decreases the odds of recovering the business by 82.34% compared to those not recovering their businesses. A unit increase of running several businesses increases the odds of recovering to the business by 80.91% compared to those not recovering their businesses.

Moreover, a unit increase of personal savings increases the odds of not recovering the business by 91.41% compared to those recovering their businesses. A unit increase of government support decreases the odds of recovering the business by 66.34% compared to those not recovering their businesses. A unit increase of family and friend’s support increases the odds of not recovering the business by 59.12% compared to those recovering their businesses. A unit increase of business continuity training increases the odds of recovering the business by 58.69% compared to those not recovering their businesses. A year increase in business operation increases the odds of recovering to the business by 95.57% compared to those not recovering their businesses.

### Sales before and after flood

Understanding the sales of MSMEs before and after the flood was one of the important items to be considered for validating recovery of business. Before flood, about 72 (35.64%) of the MSMEs mentioned that they had an income of TZS 30 000 ≤ *x* ≤ 200 000, 45 (equivalent to 22.28%) had an income of TZS 200 000 ≤ *x* ≤ 400 000, 43 (equivalent to 21.29%) had an income of TZS 400 000 ≤ *x* ≤ 600 000 and very few (2:0.99) had an income of ≥ 5 000 000 TZS Reporting on the income earned after being hit by floods, slightly significant number (80:39.6%) reported an income of TZS 0 ≤ *x* ≤ 30 000, followed by 54 (26.73%) who had income of TZS 30 000 ≤ *x* ≤ 200 000 and 30 (14.85%) with income between TZS 200 000 ≤ *x* ≤ 400 000. There were no MSMEs who reported an income of *x* > 5 000 000 TZS

The income as reported by business owners (Table 4) before flood ranged between TZS 30 000 ≤ *x* ≤ 5 000 000 but after the flood it dropped to TZS 0 ≤ *x* ≤ 4 000 000. The implication to the reported income are twofold: (1) businness owners who managed to reestablish their business were operating at loss; and (2) the fact that other MSMEs reported earning nothing (zero) after being affected by floods indicates the existence of MSMEs who could not recover their business.

**TABLE 4:** Micro, small and medium enterprises’ income before and after flood.

Income range (TZS)	Observations before flood			Observations after flood		
	Observations	Percentage (%)	Cum.	Observations	Percentage (%)	Cum.
0 ≤ *x* ≤ 29 999	0	0	0	80	39.6	39.6
30 000 ≤ *x* ≤ 200 000	72	35.64	35.64	54	26.73	66.34
200 000 ≤ *x* ≤ 400 000	45	22.28	57.92	30	14.85	81.19
400 000 ≤ *x* ≤ 600 000	43	21.29	79.21	18	8.91	90.1
600 000 ≤ *x* ≤ 800 000	7	3.47	82.67	6	2.97	93.07
800 000 ≤ *x* ≤ 1 000 000	12	5.94	88.61	3	1.49	94.55
1 000 000 ≤ *x* ≤ 2 000 000	13	6.44	95.05	6	2.97	97.52
2 000 000 ≤ *x* ≤ 4 000 000	8	3.96	99.01	5	2.48	100
< 5 000 000	2	0.99	100	-	-	-

**Total**	**202**	**100**		**202**	**100**	

TZS, Tanzanian Shilling; Cum, cumulative.

It was important to use paired *t* test (Table 5) so as to show the comparison between sales before flood and after floods. Since (*p* = 0.0000), we reject the null hyothesis at level of significant α = 5% (0.05), hence there is statistical significant diference between mean sales before floods and mean sales after floods. Mean sales before floods (560 014) were greater than mean sales after flooding (258 712).

**TABLE 5:** Paired *t* test.

Variable	Observations	Mean	s.e.	s.d.	(95% CI)	
Sales before flood	202	560014.9	56623.15	804766.1	448363.3	671666.4
Sales after flood	202	258712.9	35176.67	499954.4	189350.2	328075.5
Difference	202	301302.0	38001.04	540096.3	226370.1	376233.8

s.e., standard error; s.d., standard deviation; CI, confidence interval.

Note: mean(diff) = mean(sales before flood − sales after flood); *t* = 7.9288; Ho: mean(diff) = 0; degrees of freedom = 201; Ha: mean(diff) < 0; Ha: mean(diff) ! = 0; Ha: mean(diff) > 0; Pr(*T* < *t*) = 1.0000; Pr(|*T*| > |*t*|) = 0.0000; Pr(*T* > *t*) = 0.0000.

### Adaptation strategies

**FIGURE 1:**
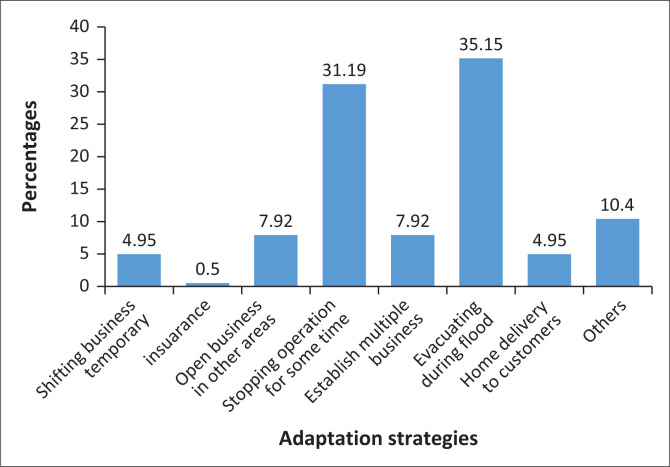
Flood adaptation strategies.

In an effort to deal with floods, MSMEs in Kigogo ward are using various strategies. As can be seen in Figure 1, the strategy mentioned by a substantial proportion (35.15%) is evacuating during floods. Evacuating is done by transferring equipment or consumables to safer areas; they do so when they feel the occurrence of heavy rain and sometimes when they receive early warning from their ward disaster management committee (WDMC). The next largest strategy is stopping doing business during flood (31.19%), and the remaining strategies were mentioned by a few.

### Business recovery support

The study findings revealed that residents doing business in Kigogo receive supports from family and friends. Ratings as shown in Table 6 show advice as among the support ranked high (44.55) and they are using that opportunity to borrow the same people without any interest as witnessed by 46.04%.

**TABLE 6:** Distribution of business support received from family member and friends.

Business support	Very high	High	Medium	Low
I receive advise from family and friends	41.09	44.55	11.88	2.48
I receive financial support from families and friends	30.20	43.56	18.32	7.92
I borrow money from family members and friends with interest	2.48	9.90	24.75	62.87
I borrow money from family members and friends without interest	46.04	24.26	21.29	8.42

Bonding social capital shown is very important in helping MSMEs to recover their businesses; if used wisely, it is an opportunity for them to be financially resilient for future disasters. Although a large percentage have been receiving advice from family and friends, it is good to note that this help is very important psychologically, especially during the flood period when they are desperate. The fact that there are MSMEs that did not recover and recovered but operating at loss was crucial to examine SWOT of the adaptation strategies.

### Strength

Micro, small and medium enterprises have great support from friends and their families where they receive advise and even support during evacuation. They also have a proactive WDMC that gives them early warning information regarding the expected rain that will probably lead to floods. They have lived in that dangerous area for a long time and so they are able to identify escape routes when a serious problem occurs.

### Weaknesses

Many of the methods used as mitigation measures (e.g. evacuation) do not have significant impact on reducing the impact of the area where they do their business. Also, many of them do business that is hand-to-mouth and thus lead to economic instability and failure to maintain savings that could enable them to continue with business profitably after disaster. No training has ever been done for them in relation to how to deal with floods and so they protect themselves by experience.

### Threat

**FIGURE 2:**
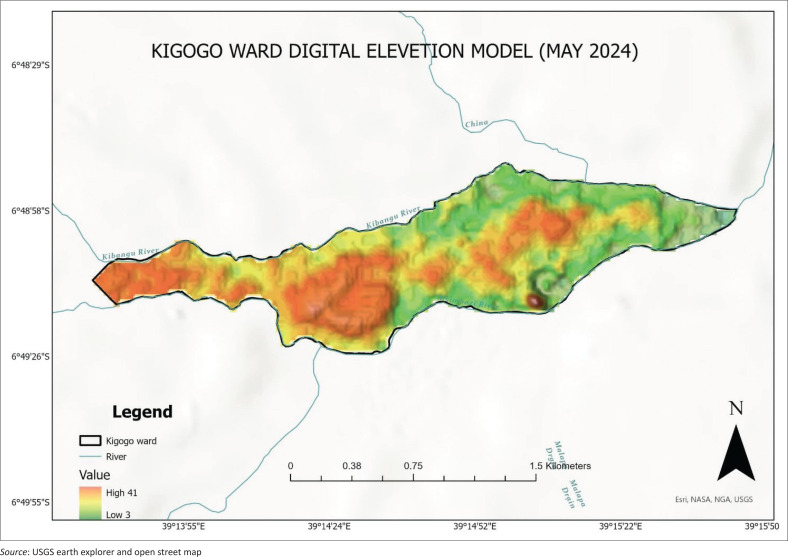
Digital elevation model of the Kigogo River surroundings.

Kigogo is vulnerable to frequent floods given its location (surrounded by rivers in all sides; Figure 2), so the effects of floods are inevitable. Many businessmen also lack understanding regarding appropriate mitigation strategies, especially what to do so as to mitigate flood impact on their business. The majority of MSMEs own only one business, which is a threat to their income, especially when they fail to continue operations after being faced with floods.

### Opportunities

They have a strong WDMC that normally gives them information regarding possibility of flooding. If they were to use this information well, it would help them in taking long-term mitigation measures. The fact that they reported borrowing from banks and others is an ample opportunity that they are capable of borrowing. The implication is that they are able to raise their capital and be able to borrow for the purpose of investing in mitigation measures. The majority have lived in Kigogo for a considerable number of years, and thus they are aware of floods.

## Discussion

The study found strong empirical evidence that the majority of businesses in Kigogo ward are dominated by MSMEs. Consistent with literature, this study established that MSMEs play an important role by contributing significantly in the employment sector. Although most of the employees are casual labourers who work and are paid daily, what is important to say is that at least they get a chance to work and receive a wage that helps them survive. In addition to providing employment to many people, behind these MSMEs, there are many family members who depend on them for education, food, treatment and shelter. On that account, it is important that MSMEs continue to exist because their contribution to helping the nation reduce the problem of unemployment is enormous.

Although all MSMEs interviewed have their businesses in Kigogo, in flood-prone areas, not all experienced floods alike. One of the reasons is the type of business they do; according to χ^2^ test, it was revealed that MSMEs with retail shops suffered more damage to the business area and damage to equipment. Micro, small and medium enterprises involved in food-related business suffered the effects of loss of properties and/or goods. For others who engage in service-related business, χ^2^ did not show any association.

Many MSMEs admitted that they are severely affected by floods. They implement various adaptation strategies, and majority adopted the strategy of evacuation during floods. Others opted to stop operation for some time. The major factor that made MSMEs to opt the mentioned strategies is presence of stagnant water. The strategies they used are an indication that they failed to sell, a finding that is consistent with the information they have given about the losses they are experiencing during flood. As stated in literature, frequent floods normally occur in low-lying areas that are unorganised, and same areas are preferred by many (including MSMEs). They are exposed to floods and most residents are not involved in investing in adaptation strategies. Owing to the continued presence of climate change, floods are expected to increase rapidly and also the damage they cause is also expected to heighten. Many areas that are subject to frequent floods are often unorganised and therefore make small-scale businesses operate in a difficult and vulnerable environment (Lundy et al. 2017).

Analysis done through logistic regression enabled identifying factors that influence business recovery. Previous scholars reported various factors that determine business recovery (Chang et al. 2022; Khan & Sayem 2013; Pathak & Ahmad 2016; Paul et al. 2014), which are somewhat incosistent with the findings of this study. The differences observed in the outcome are because of the nature of business (majority investigated MSEs) and types of disaster.

## Conclusion

Consistent with the literature, findings of this study indicate that MSMEs in flood-prone areas are tremendously vulnerable to floods, to the extent that other MSMEs failed to recover or recovered but operated at loss while their presence is very crucial. The presence of MSMEs is very important because they are major contributors to the economy and they help in reducing the employment problem that has become a challenge for many middle and developing countries. As the study showed, MSMEs in Kigogo need to be flood-resilient so that they can continue to contribute to the national economy and to the economy of individuals in particular.

The study findings present several practical implications. First and foremost, they suggest that disaster mitigation is compelling for the recovery of MSMEs. Consequently, MSMEs should be conscious of flood mitigation measures as they contribute to disaster recovery. However, the question is: how will they succeed? According to Asgary (2012) and Twerefou et al. (2019), training and education before and after disasters are very critical for the recovery of MSMEs. Therefore, pertinent training would empower MSMEs to improve the way they deal with floods to minimise risks to their businesses.

Although the research results contribute to the existing literature, especially when considering that this is the first study on business recovery of MSMEs in flood-prone areas in Dar es Salaam, the results cannot be generalised. Future studies should be conducted in other flood-prone areas in Dar es Salaam to ascertain if the factors affecting business recovery in Kigogo are similar to those in other areas affected by floods.
